# Molecular and Functional Analysis of UDP-*N*-Acetylglucosamine Pyrophosphorylases from the Migratory Locust, *Locusta migratoria*

**DOI:** 10.1371/journal.pone.0071970

**Published:** 2013-08-19

**Authors:** Xiaojian Liu, Feng Li, Daqi Li, Enbo Ma, Wenqing Zhang, Kun Yan Zhu, Jianzhen Zhang

**Affiliations:** 1 Research Institute of Applied Biology, Shanxi University, Taiyuan, Shanxi, People’s Republic of China; 2 State Key Laboratory of Biocontrol, School of Life Sciences, Sun Yat-sen University, Guangzhou, Guangdong, People’s Republic of China; 3 Department of Entomology, Kansas State University, Manhattan, Kansas, United States of America; National Cancer Institute, United States of America

## Abstract

UDP-*N*-acetylglucosamine pyrophosphorylases (UAP) function in the formation of extracellular matrix by producing *N*-acetylglucosamine (GlcNAc) residues needed for chitin biosynthesis and protein glycosylation. Herein, we report two *UAP* cDNA’s derived from two different genes (*LmUAP1* and *LmUAP2*) in the migratory locust *Locusta migratoria*. Both the cDNA and their deduced amino acid sequences showed about 70% identities between the two genes. Phylogenetic analysis suggests that *LmUAP1* and *LmUAP2* derive from a relatively recent gene duplication event. Both *LmUAP1* and *LmUAP2* were widely expressed in all the major tissues besides chitin-containing tissues. However, the two genes exhibited different developmental expression patterns. High expression of *LmUAP1* was detected during early embryogenesis, then decreased greatly, and slowly increased before egg hatch. During nymphal development, the highest expression of *LmUAP1* appeared just after molting but declined in each inter-molting period and then increased before molting to the next stage, whereas *LmUAP2* was more consistently expressed throughout all these stages. When the early second- and fifth-instar nymphs (1-day-old) were injected with *LmUAP1* double-stranded RNA (dsRNA), 100% mortality was observed 2 days after the injection. When the middle second- and fifth-instar nymphs (3- to 4-day-old) were injected with *LmUAP1* dsRNA, 100% mortality was observed during their next molting process. In contrast, when the insects at the same stages were injected with *LmUAP2* dsRNA, these insects were able to develop normally and molt to the next stage successfully. It is presumed that the lethality caused by RNAi of *LmUAP1* is due to reduced chitin biosynthesis of the integument and midgut, whereas *LmUAP2* is not essential for locust development at least in nymph stage. This study is expected to help better understand different functions of *UAP1* and *UAP2* in the locust and other insect species.

## Introduction

*N*-acetylglucosamine (GlcNAc), a substrate required for glycosylphosphatidylinositol (GPI) anchor formation, is an important component of glycosyl groups that modify extracellular proteins [1]. GlcNAc also contributes to the structure and function of various extracellular matrix. Chitin is an linear biopolymer of the sugar *N*-acetylglucosamine (GlcNAc) connected by β-1,4-linkages. In insects, chitin is an integral part of cuticle, trachea and peritrophic matrix (PM) [2], [3]. Insects must consistently synthesize and degrade chitin to allow ecdysis and regeneration of the PM. However, current knowledge on insect chitin biosynthesis is still fragmentary.

**Figure 1:**
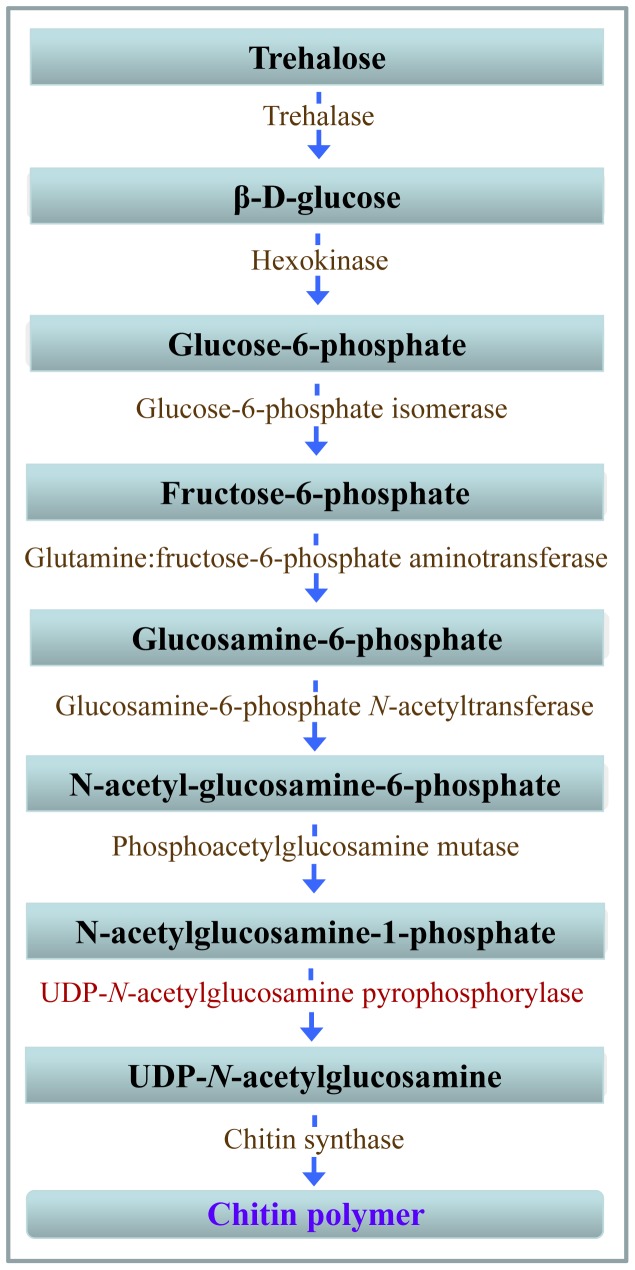
A brief diagram of chitin biosynthetic pathway in insects. The diagrammatic representation is based on Merzendorfer and Zimoch (2003). The substrates are shown in black on blue bars. The UDP-*N*-acetylglucosamine pyrophosphorylase encoded by *LmUAP1* or *LmUAP2* is shown in red and other enzymes are in brown.

Generally, chitin biosynthetic pathway begins with trehalose, and consists of at least eight key enzymes (Fig. 1) [3]–[5]. Many previous studies focus on the first enzyme trehalase and the last enzyme chitin synthase. Chitin synthase converts UDP-*N*-acetylglucosamine (UDP-GlcNAc) to the growing chitin polymer [6]. UDP-GlcNAc is the active form of GlcNAc and its formation is catalyzed by UDP-GlcNAc pyrophosphorylase (UAP) in the cell cytoplasm via the following reversible reaction: UTP+GlcNAc-1-P ↔UDP-GlcNAc+ppi [7]. UAP is also important for glycosylation of proteins, sphingolipids and secondary metabolites with *N*-acetylglucosamine (GlcNAc) or GPI anchors which bridge the proteins to cell membrane, or for conjugation of 7-β-hydroxylated bile acids [8], [9].

UAP is widely distributed in nature and has been partially purified from bacteria, yeast, calf liver and sheep brain [10]–[12]. It has been purified to homogeneity from pig hepatocytes and various properties of the enzyme were determined [13]. The genes encoding yeast and human UAPs were isolated and sequenced [14]. Later, researchers found that both the human UAP1 isoforms, UAPA and UAPB (also called AGX1 and AGX2), can use GlcNAc-1-P or *N*-acetylgalactosamine (GalNAc-1-P) as substrates, although the former is usually the preferred substrate [7]. The *UAP* cDNAs have also been identified from insects whose genomes have been completely sequenced, which include *Drosophila melanogaster*, *Anopheles gambiae*, *Aedes aegypti*, *Tribolium castaneum, Bombyx mori*, *Apis mellifera* and *Acyrthosiphon pisum* [15]. Most insect species have only one *UAP* gene except for *T. castaneum*, which has been known to possess two different *UAP* genes (*TcUAP1* and *TcUAP2*). Both the cDNA and their deduced amino acid sequences of *TcUAP1* and *TcUAP2* share the identities of about 60%. RNA interference (RNAi) experiments demonstrate that both *TcUAP* genes are critical for insect survival, but only *TcUAP1* is required for chitin biosynthesis of the cuticle and PM. RNAi of *TcUAP2* also led to high mortality, but it may be due to defective glycosylation of proteins or secondary metabolites, whose functions are critical for insect survival [15].

The importance of UAP for insect development has also been shown by studies on *D. melanogaster*. Mutants of *DmUAP* (also called *mummy*, *cabrio* or *cystic* in this species) show many defects ranging from trachea morphogenesis, cuticle formation, central nervous system fasciculation and dorsal closure to eye development [16], [17]. Most of the cuticle or tracheal morphogenesis defects can be attributed to the reduced chitin content, whereas others such as fasciculation defects and eye development abnormalities are likely due to defective glycosylation of proteins.

*Locusta migratoria*, a member of orthopterans, is one of the most destructive agricultural.

pests in the world. In China, extensive outbreaks of locusts have been increasing in recent decades, which are associated with locust’s long-distance migration and resistance to pesticides [18]. *L. migratoria* is a typical hemimetabolous insect; it develops from egg to nymph and then directly to adult without going through pupal stage. However, there is little information on chitin biosynthesis in this insect. Our previous studies showed that silencing of two chitin synthase genes (*LmCHS1* and *LmCHS2*) by RNAi led to high mortality of nymphs [19], [20]. We further demonstrated that there was a specialization in the function of each of the two chitin synthase genes. Suppression of *LmCHS1* expression by RNAi disrupted the molting process whereas suppression of *LmCHS2* affected the formation of well-structured PM and ceased insect feeding. In this paper, we report two *UAP* genes (*LmUAP1* and *LmUAP2*) from *L. migratoria*. To our knowledge, this is the second insect species after *T. castaneum* known to possess two different *UAP* genes. Our study consisted of: 1) identification and sequencing of two full-length cDNAs of *LmUAP1* and *LmUAP2*, 2) analysis of expression patterns of the two genes in different tissues and at different developmental stages, and 3) functional analysis of the two genes by RNAi.

## Materials and Methods

### Insects Rearing

*Locusta migratoria* were obtained from Insect Protein Co., Ltd. of Cangzhou City in China and reared using fresh wheat seedlings and bran in a growth chamber (Yiheng, China) at 30±2°C and 40±10% relative humidity (RH) with a 14∶10-h light: dark photoperiod. Insects of different developmental stages were synchronized for experiments. Adults were allowed to lay eggs and the eggs from the same day were then incubated in a growth chamber under the same rearing conditions.

### Identification and Sequencing of *LmUAP1* and *LmUAP2* cDNAs

By searching the *L. migratoria* transcriptome database, two cDNA sequences apparently derived from two different genes encoding UAPs (i.e., *LmUAP1* and *LmUAP2*) were identified. To confirm the predicted coding sequences of the two genes, gene-specific primers were used to amplify cDNAs by reverse transcription PCR (RT-PCR) using cDNA templates prepared from the whole insect body. PCR products were purified by 1% agarose gel, subcloned into pGEM-T easy vector (Promega, Madison, WI), and sequenced in both directions by Beijing Aoke Biotechnology Co., Ltd. (Beijing, China).

### Analyses of *LmUAP1* and *LmUAP2* cDNA and Deduced Amino Acid Sequences

The translation of cDNA sequences into amino acid sequences was performed using ExPASy Proteomics website (http://cn.expasy.org/). The similarity search and homology comparisons of the two *LmUAP* deduced amino acid sequences were made with other insect UAP sequences using the blastp tool at NCBI website (http://www.ncbi.nlm.nih.gov/). The deduced amino acid sequences of *LmUAP1* and *LmUAP2* and *TcUAP1* and *TcUAP2* were aligned by using GeneDoc software (http://www.nrbsc.org/gfx/genedoc/). The substrate binding sites were also predicted using the programs at NCBI website. The molecular mass, isoelectric point (pI), signal peptide and transmembrane regions were predicted based on their amino acid sequences from ExPASy Proteomics website.

### Phylogenetic Analysis of UAP Proteins

Multiple amino acid sequence alignment of known UAPs was carried out using ClustalW software [21]. Values for multiple alignment were gap opening penalty 10, gap extension penalty 0.2, and delay divergent sequences 30%. Protein weight matrix was set at gonnet series. The phylogenetic tree was constructed by MEGA 5. The unweighted pair group method with arithmetic mean (UPGMA) method was used with poisson correction for multiple amino acid substitution and with 1000 random bootstrap replicates.

### Analyses of Tissue and Embryo Development Expression Patterns of *LmUAP1* and *LmUAP2*

To analyze the tissue-dependent expression patterns, a total of 10 different tissues including integument, foregut, midgut, hindgut, gastric caeca, Malpighian tubules, fatbodies, muscles, wing and trachea were dissected from fifth-instar nymphs (2-day-old) of *L. migratoria*. The nymphs were chilled on ice and tissues were carefully separated and immediately placed in liquid nitrogen. At least 10 nymphs were collected to dissect the tissues in one replicate with three independent biological replications. Total RNA from each sample was isolated using RNAiso™ Plus (TaKaRa, Japan) according to the protocol, then analyzed on 0.8% agarose gel to ensure their integrity. The final concentration was measured using SpectraMax 190 microplate reader and SOFTmax software (Molecular Devices, USA). Aliquots of 50 µg of total RNA were then treated with RNase-free DNase (TaKaRa) and first-strand cDNA was synthesized using M-MLV reverse transcriptase (TaKaRa) following the manufacturer’s instruction.

To analyze embryo development-dependent expression patterns, newly laid eggs from the same day were incubated in a growth chamber and collected at 24 h intervals during the embryonic development. At least 20 eggs were used in each replicate, and three replicates were collected. To analyze nymphal molting-dependent expression patterns of *LmUAP1* and *LmUAP2*, the integument was dissected in each day from fourth-instar nymphs to adults. A pool of three insects was randomly collected as a biological replication with three independent biological replications. Isolation of total RNA and synthesis of first-strand cDNA were performed as described above.

### Identification and Validation of Reference Genes under Different Conditions

To obtain reliable reverse transcription-quantitative PCR (RT-qPCR) data, six commonly used reference genes were obtained from the *L. migratoria* transcriptome database. The selected genes were *β-actin*, *EF1α, GAPDH*, *RP49*, *α-Tubulin*, and *18SrRNA*. The primer information of each gene is shown in Table S1. We analyzed the expression of these genes in different tissues and developmental stages by employing RT-qPCR. The reaction mixture contained 2 µL of a 10-fold diluted cDNA, 0.4 µM of each primers and SYBR Premix Ex Taq (2x, Toyobo, Japan) in a total volume of 20 µL. RT-qPCR was performed on a 7300 Real-Time PCR system (Applied Biosystems, USA). The optimized RT-qPCR program consisted of initial denaturation at 95°C for 10 s, followed by 40 cycles of 95°C for 5 s and 60°C for 34 s. The temperature was increased from 60 to 95°C at a rate of 2°C/min, and the fluorescence was detected every 15 s to construct the melting curve. In addition, a no-template control (NTC) was included for each primer pair. The amplification efficiency of each gene was calculated from the slope of the log-linear portion of the curve generated by amplification from serially diluted cDNAs. The RT-qPCR for each gene was performed with three biological replications, each with two technical measurements. The 2^−ΔCT^ method was used to calculate the relative transcript level of each reference gene. Two software programs, geNorm [22] and NormFinder [23] were applied to assess the stability of these genes. The optimal number of reference genes for effective normalization was calculated by the geNorm software.

### Analyses of Expression Patterns of *LmUAP1* and *LmUAP2*

Based on the nucleotide sequence alignment between *LmUAP1* and *LmUAP2*, specific primers (Table 1) were designed for detecting transcription level of each gene. RT-qPCR for each gene was performed with three biological replications, each with two technical measurements. The amplification efficiency of each gene was calculated from the slope of the log-linear portion of the curve generated by amplification from serially diluted cDNAs. The 2^−ΔΔCT^ method was used to calculate the relative transcript levels of *LmUAP1* and *LmUAP2* in different tissues and developmental stages.

**Table 1:** Primers used to amplify cDNA sequences, analyze the expression profiles and synthesize dsRNA of both *LmUAP1* and *LmUAP2* genes.

Application of primers	Gene name	Primer name	Primer sequence (5′–3′)	Product size (bp)
cDNA cloning	*LmUAP1*	UAP1F	ACTGTGTTAGCACAGTTTCAGAATG	1483
		UAP1R	AGAAATTTAGCTTTGTTCACTAGGA	
	*LmUAP2*	UAP2F	ACCGATCACTATGCAGGATCTTATA	1496
		UAP2R	CATTTCAGCAATAATTATTTTCTTG	
RT-qPCR analysis	*LmUAP1*	EUAP1F	TACGGGACCGTAAGGTGTTGG	139
		EUAP1R	CCACATTCTGCATTTTTGCTTATAC	
	*LmUAP2*	EUAP2F	GTACCTAAATGCTCATGGTGTGGAT	154
		EUAP2R	GTCCACCTGGCAAACAACTCCT	
	*β-actin*	β-actinF	CGAAGCACAGTCAAAGAGAGGTA	156
		β-actinR	GCTTCAGTCAAGAGAACAGGATG	
dsRNA synthesis	*LmUAP1*	dsUAP1F	TAATACGACTCACTATAGGGGCAAAAATGCAGAATGTGGA	368
		dsUAP1R	TAATACGACTCACTATAGGGCCGCAAATTGAAAGACATCA	
	*LmUAP2*	dsUAP2F	TAATACGACTCACTATAGGGAGCAGAGGAATGCTGATGGT	499
		dsUAP2R	TAATACGACTCACTATAGGGGAGCGCAAATGACATGGAGA	
	*GFP*	dsGFPF	TAATACGACTCACTATAGGGGTGGAGAGGGTGAAGG	712
		dsGFPR	TAATACGACTCACTATAGGGGGGCAGATTGTGTGGAC	

### Analysis RT-qPCR Data

The relative expression of each gene was calculated as fold changes as compared with the lowest expression among different tissues or developmental stages. The most stably expressed combination of reference genes was selected for RT-qPCR data normalization. Results were expressed as mean ± SD of three biological replications. The means among different tissues or developmental stages were compared using Tukey’s HSD multiple comparisons with SPSS 15.0 software (SPSS Inc., Chicago, IL). Means with *P*<0.05 were considered to be significantly different.

### Functional Analysis of *LmUAP1* and *LmUAP2*

The most divergent nucleotide sequences of *LmUAP1* and *LmUAP2* were chosen as target regions for synthesizing gene-specific double-stranded RNA (dsRNA) to study the functions of *LmUAP1* and *LmUAP2*. Pairs of forward and reverse primers with T7 RNA promoter sequence at the 5′-end were synthesized within these regions (Table 1). The dsRNA of *GFP* was used to serve as a negative control for monitoring possible non-specific effect of dsRNA molecules. PCR was performed to prepare cDNA template from the whole body of fifth-instar nymphs for dsRNA synthesis. PCR products were subcloned and sequenced to confirm the specificity of each gene. The dsRNA for each gene was synthesized by T7 RiboMAX™ Express RNAi System (Promega) according to the manufacturer’s instruction using the sequence-verified plasmids as templates. Each dsRNA was dissolved in appropriate volume of nuclease-free water and analyzed on 1.5% agarose gel to ensure their integrity. The final concentration of dsRNA was adjusted to 1.5 µg/µL using SpectraMax 190 microplate reader and SOFTmax software (Molecular Devices, USA).

To determine whether *LmUAP1* and *LmUAP2* transcripts are essential for early developmental time in nymphal stages, the second- and fifth-instar nymphs on day 1 were selected for the RNAi experiments. Aliquots of 3 µg (for second-instar nymphs) or 10 µg (for fifth-instar nymphs) *LmUAP1* or *LmUAP2* dsRNA were injected into the dorsal side between the second and third abdominal segments of locusts using a manual microinjector (Ningbo, China). The equivalent amounts of *GFP* dsRNA were used as controls. After the injections, nymphs were maintained under the same conditions as previously described and observed for any visible abnormalities or mortality.

For observing morphological changes, 40 second- and 55 fifth-instar nymphs were used in this experiment. For gene expression analysis, three biological replicates, each with three insects, were used. The transcript levels of both *LmUAP* genes were measured at 24 h and 48 h after the injection of *LmUAP1* or *LmUAP2* dsRNA. Briefly, total RNA was isolated from the whole bodies of each sample. The down-regulation of *LmUAP1* or *LmUAP2* transcript was determined by RT-qPCR as described above.

To determine whether *LmUAP1* and *LmUAP2* were also essential for late nymphal development, we also injected *LmUAP1* or *LmUAP2* dsRNA in second- and fifth-instar nymphs on day 3–4. The observation for the phenotype and the evaluation of the silencing efficiency for each target gene were the same as described above.

## Results

### Characterization of Two *UAP* cDNAs and their Deduced Amino Acid Sequences

Our search of the *L. migratoria* transcriptome database revealed two cDNAs (*LmUAPs*) putatively encoding two different UDP*-N-*acetylglucosamine pyrophosphorylases. The two cDNA fragments with sizes of about 1.4 kb were amplified by RT-PCR using forward primers that contained the presumed start codon and reverse primers with 5′-end complementary to the stop codon. Our sequencing results confirmed the identities of *LmUAP* cDNAs. They were, therefore, named *LmUAP1* (GenBank accession number JX484802) and *LmUAP2* (GenBank accession number JX484803).

**Figure 2:**
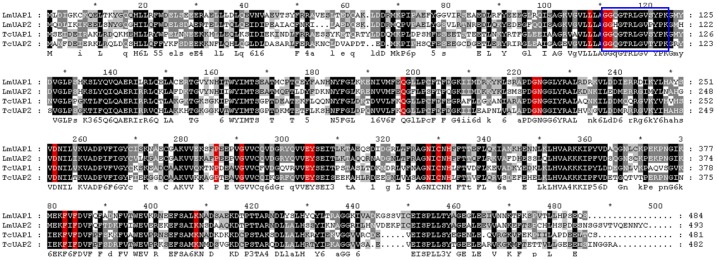
Alignment of deduced amino acid sequences of *LmUAP1* and *LmUAP2* from *Locusta migratoria* and *TcUAP1* and *TcUAP2* from *Tribolium castaneum* using GeneDoc software.

The full-length cDNA sequence of *LmUAP1* is 2223 bp with an open reading frame (ORF) of 1455 bp and a 66-nucleotide 5′-untranslated region (UTR) and a 702-nucleotide 3′-UTR, which encodes a protein of 484 amino acid residues. Its predicted molecular mass and pI of the deduced protein are approximately 55.1 kDa and 5.74, respectively. The full-length cDNA of *LmUAP2* is 1995 bp with the ORF of 1482 bp and a 158-nucleotide 5′-UTR and a 355-nucleotide 3′-UTR, which encodes a protein of 493 amino acid residues. The calculated molecular mass and pI of the predicted protein are approximately 55.8 kDa and 5.64, respectively. Our predictions did not reveal any signal peptide and transmembrane regions. Further analysis showed that the identities between *LmUAP1* and *LmUAP2* were 70% for both the nucleotide and deduced amino acid sequences (Fig. 2). The conserved substrate binding sites of 17 amino acid residues were found in both LmUAPs and TcUAPs. The signature sequence of UAPs, GGXXTXXGXXXPK, was also identified in LmUAP1 and LmUAP2, where X presents any of amino acid residues.

### Phylogenetic Analysis of UAPs

**Figure 3:**
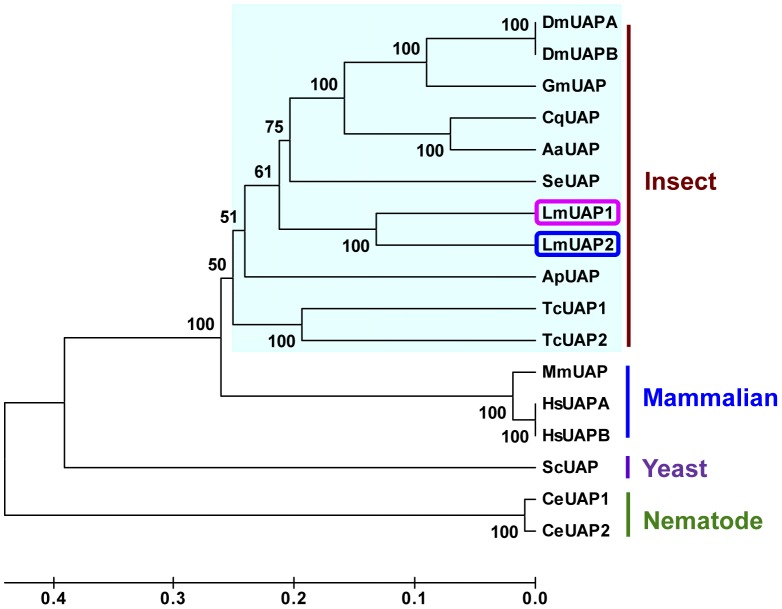
Phylogenetic analysis of UAPs in insects and other organisms. The tree was constructed based on the full-length amino acid sequences of UAPs. The scale at the bottom is in units of amino acid substitutions per site. The proteins of insects are highlighted in yellow. GenBank accession numbers are as follows: DmUAPA, *Drosophila melanogaster* (NP_609032, isoform A); DmUAPB, *Drosophila melanogaster* (NP_723183, isoform B); AaUAP, *Aedes aegypti* (EAT47260); CqUAP, *Culex quinquefasciatus* (EDS38218); BmUAP, *Bombyx mori* (BGIBMGA001609-PA); ApUAP, *Acyrthosiphon pisum* (XP_001944680); TcUAP1, *Tribolium castaneum* (NP_001164533); TcUAP2, *Tribolium castaneum* (NP_001164533); MmUAP, *Mus musculus* (BC017547); SeUAP, *Spodoptera exigua* (ACN29686); HsUAPA, *Homo sapiens* (NP_003106, isoform A); HsUAPB, *Homo sapiens* (Q16222.3, isoform B); ScUAP, *Saccharomyces cerevisiae* (NP_010180.1); CeUAP1, *Caenorhabditis elegans* (NP_497777); CeUAP2, *Caenorhabditis elegans* (NP_500511.2).

A phylogenetic tree was generated using the full-length amino acid sequences of UAPs from mammalian, insect, nematode, and yeast species. As expected, the two *L. migratoria* UAPs are tightly clustered together in the phylogenetic tree (Fig. 3). The UAPs from all the insect species first clustered and then clustered with those of other organisms.

### Identification and Validation of Reference Genes under Different Conditions

Taking advantage of the sequences available in the *L. migratoria* transcriptome database, we selected the genes encoding β-actin, EF1α, GAPDH, RP49, α-Tublin, and 18SrRNA to identify the most stably expressed reference gene under different conditions. No amplification of fluorescent signal was detected in all negative control samples, indicating that the DNase treatment effectively removed genomic DNA from all RNA samples. The primer efficiency test showed that the amplification efficiency of each gene was above 92% (Table S1). According to geNorm, two genes are required to normalize the target gene under different conditions. In different tissues of the fifth instar nymphs (2-day-old), *GAPDH* and *EF1α* were the most stably expressed genes by using the software programs geNorm and Normfinder. During the developmental stages, geNorm indicated that *RP49* and *EF1a* were the most stable genes, followed by *β-actin*, while Normfinder indicated that *β-actin* was the best candidate for normalization, followed by *EF1a*. Thus, *β-actin* and *GAPDH* were the best candidates for normalization in the RNAi study (Figures S1, S2 and S3).

### Tissue-Dependent Expression Patterns of *LmUAP1* and *LmUAP2*

**Figure 4:**
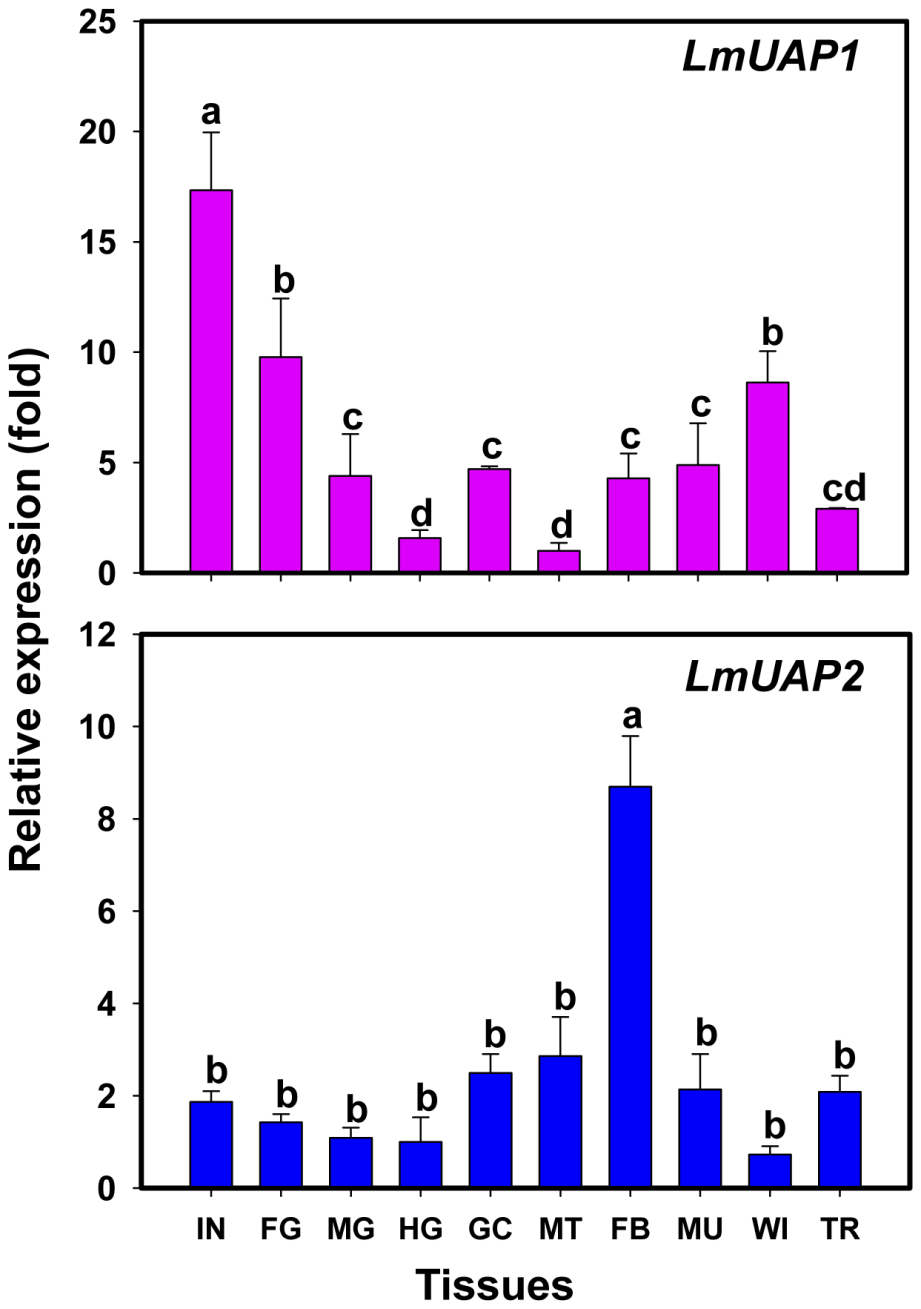
Tissue-dependent expression of *LmUAP1* and *LmUAP2* in fifth-instar nymphs on day 1 by RT-qPCR. The genes encoding EF1α and GAPDH were used as internal controls. The tissues include integument (IN), foregut (FG), midgut (MG), hindgut (HG), gastric caeca (GC), Malpighian tubules (MT), fatbodies (FB), muscle (MU), wing (WI) and trachea (TR). Data are expressed as means ± SD of three biological replications. The relative expression is shown as fold changes as compared with the tissue showing the lowest expression which is ascribed an arbitrary value of 1. Different letters on the bars of the histogram indicate significant differences among different tissues (*P*<0.05, Tukey’s HSD test; n = 3).

The primer efficiency test showed that the amplification efficiencies of *LmUAP1* and *LmUAP2* genes were 95.4 and 96.6%, respectively. The expression patterns of the two *LmUAP* genes were evaluated in 10 different tissues dissected from fifth-instar nymphs and normalized with *EF1α* and *GAPDH* by using RT-qPCR (Fig. 4). *LmUAP1* and *LmUAP2* were widely expressed in all the tissues examined. *LmUAP1* was expressed at highest level in integument, and high in foregut and wing, but low in midgut, gastric caeca, hindgut, Malpighian tubules, fatbodies, muscles and trachea. However, *LmUAP2* was expressed significantly higher in fatbodies than in other tissues examined.

### Developmental Expression Patterns of *LmUAP1* and *LmUAP2*

**Figure 5:**
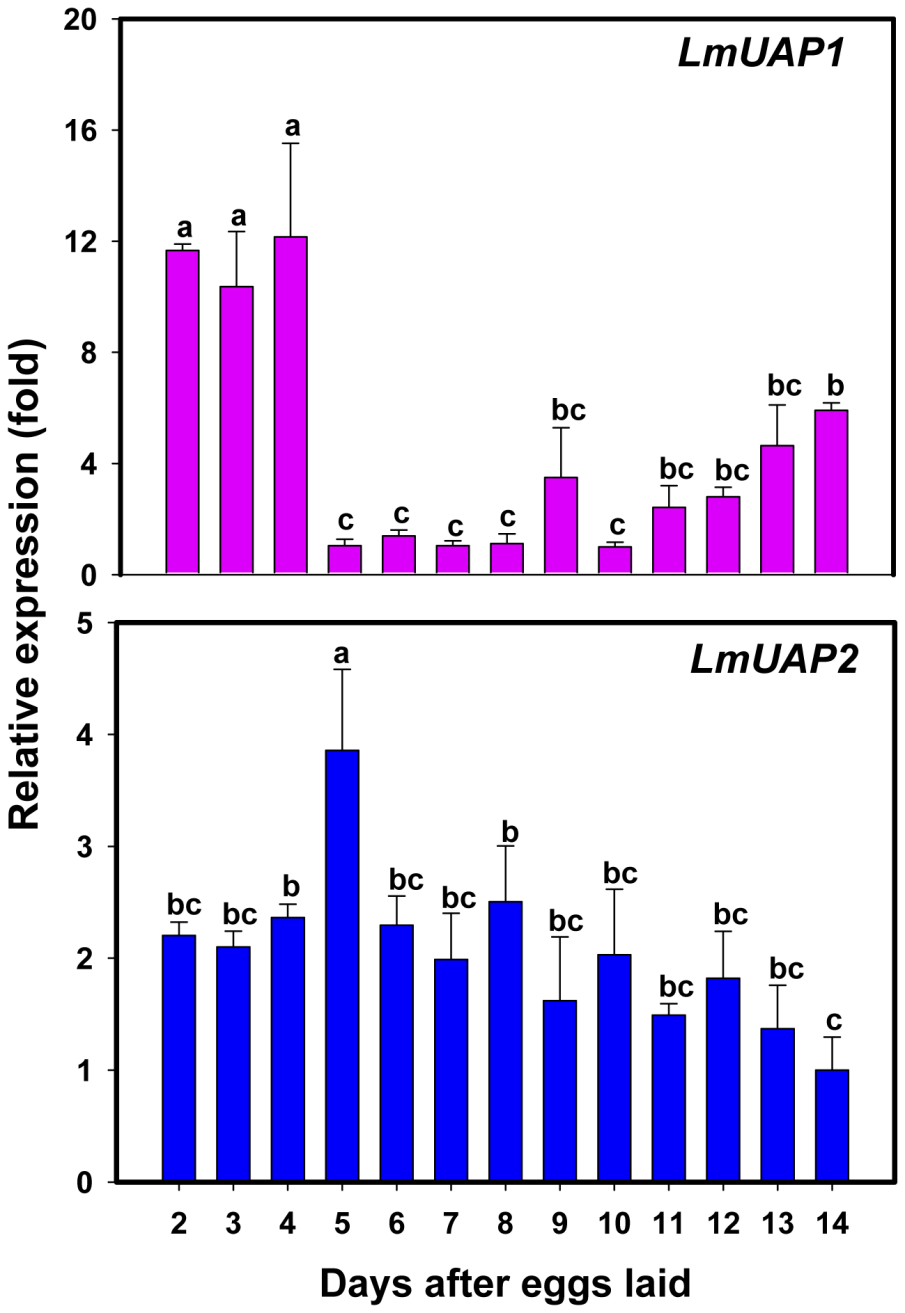
The relative expression level of *LmUAP1* and *LmUAP2* during embryonic development of *L. migratoria* by RT-qPCR. The genes encoding EF1α and β-actin were used as internal controls. Data are expressed as means ± SD of three biological replications. The relative expression is shown as fold changes as compared with the day showing the lowest expression which is ascribed an arbitrary value of 1. Different letters on the bars of the histogram indicate significant differences among different days during embryonic development (*P*<0.05, Tukey’s HSD test; n = 3).

Developmental expression patterns of the two *LmUAP* genes were evaluated in eggs, fourth and fifth-instar nymphs, and adults by using RT-qPCR. The genes encoding β-actin and EF1α were used to normalize the data. The relative transcript levels of *LmUAP1* and *LmUAP2* varied substantially throughout the developmental stages. The expression of *LmUAP1* was significantly higher during the early embryogenesis (days 2–4) than the rest of the embryogenetic stages (Fig. 5). For *LmUAP2*, the expression was relatively consistent during the embryogenesis except for the 5-day-old eggs, in which the expression was 1.5- to 3-fold higher than those in other days.

**Figure 6:**
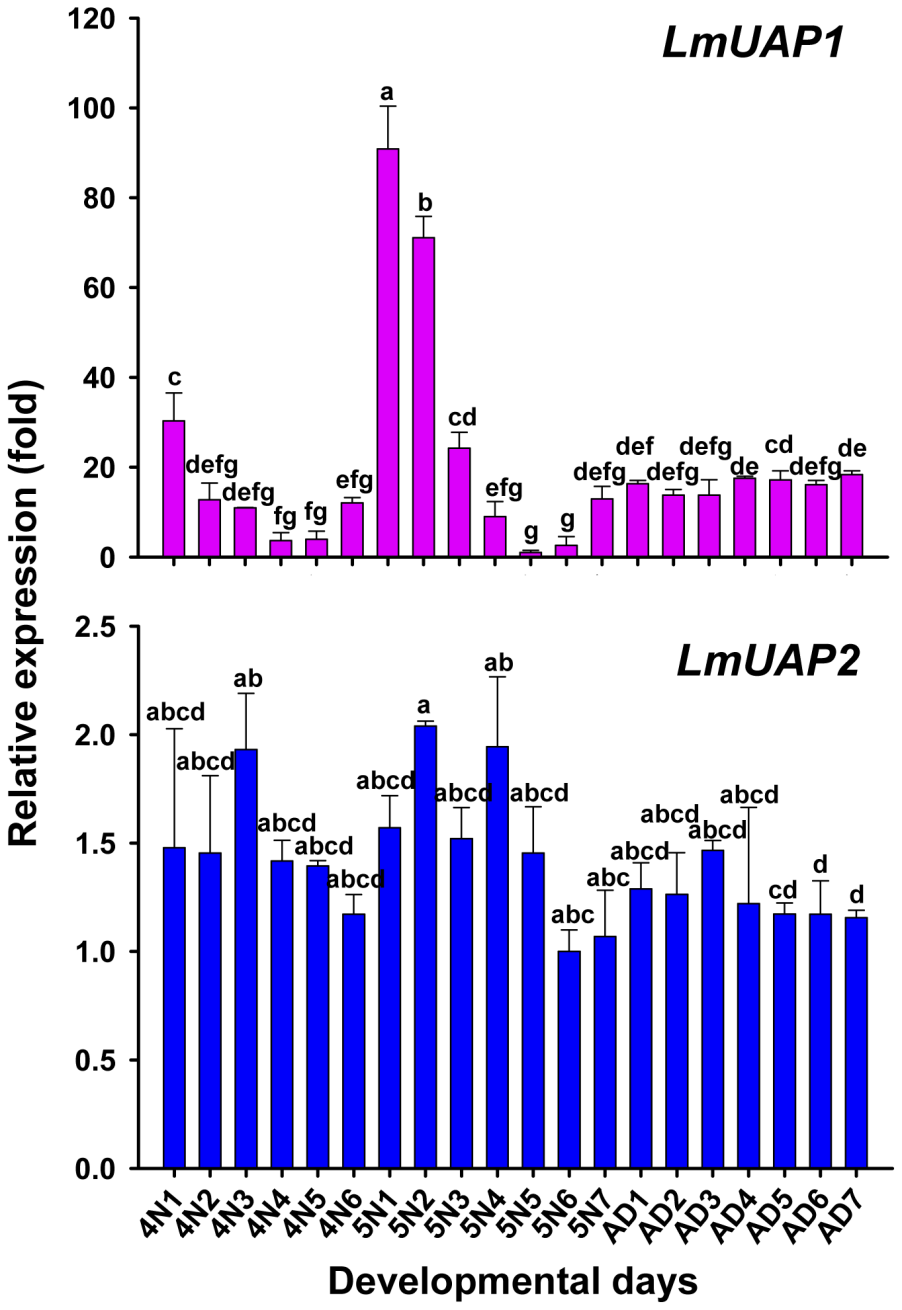
The relative expression level of *LmUAP1* and *LmUAP2* during the fourth-instar nymph to adults of *L. migratoria* by RT-qPCR. The time points were abbreviated as 4N1–4N6, 5N1–5N7 and AD1–AD7, respectively. The genes encoding EF1α and β-actin were used as internal controls. Data are expressed as means ± SD of three biological replications. The relative expression is shown as fold changes as compared with the day showing the lowest expression which is ascribed an arbitrary value of 1. Different letters on the bars of the histogram indicate significant differences among different days during development (*P*<0.05, Tukey’s HSD test; n = 3).

Because UAP is an important enzyme for chitin biosynthesis, and cuticular exoskeleton is one of the chitin-containing tissues, developmental expression patterns of *LmUAP* were investigated using the integuments of fourth- and fifth-instar nymphal and adult stages (Fig. 6). For *LmUAP1*, the highest expression was found just after fourth-instar nymph molting, but the expression gradually declined and there was no significant changes when fifth-instar nymphs molted to adults. Unlike the expression patterns of *LmUAP1*, the expression level of *LmUAP2* was relatively consistent during these stages (Fig. 6).

### Functional Analysis of *LmUAP1* and *LmUAP2*

**Figure 7:**
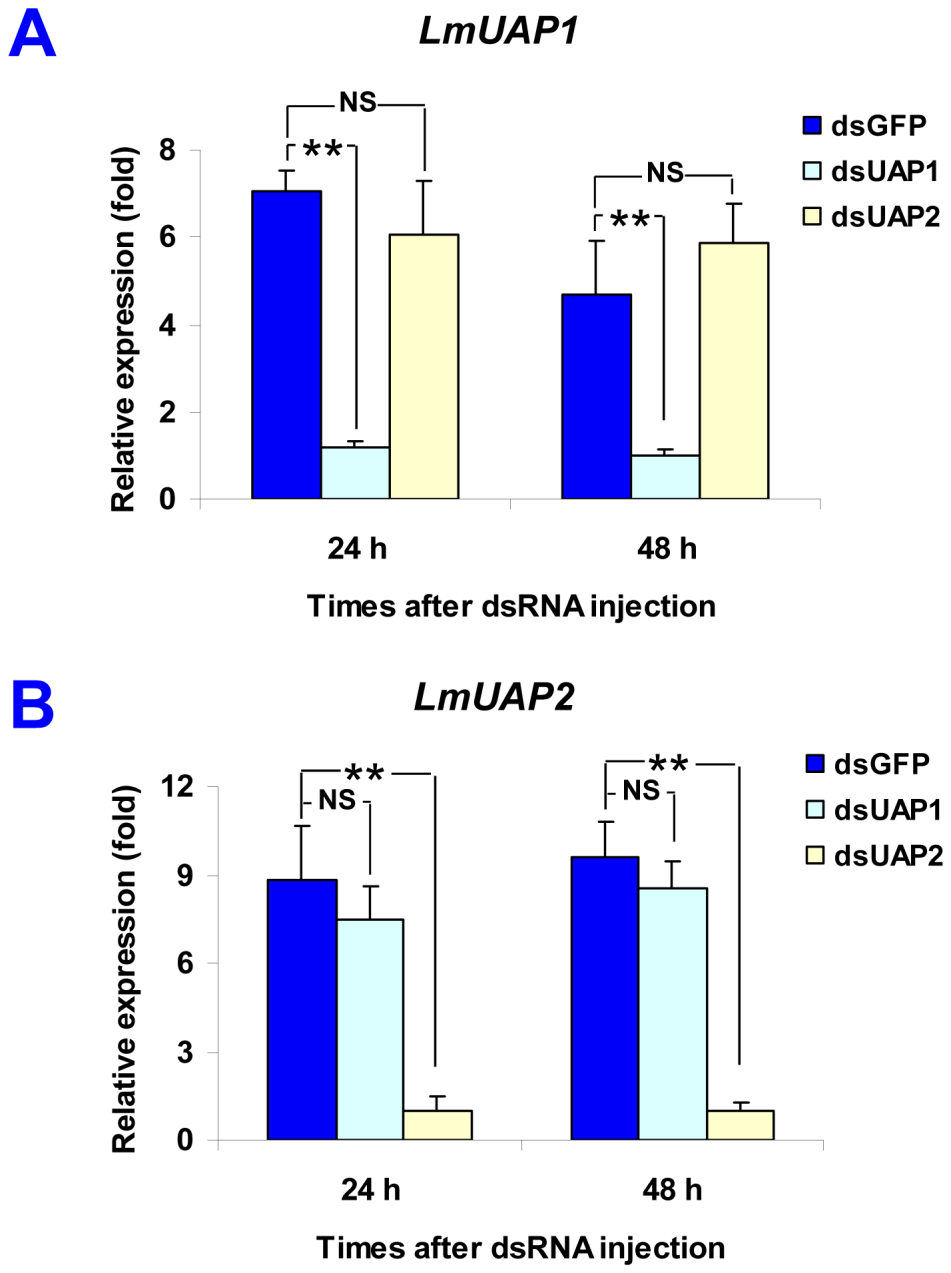
Effect of *LmUAP1* and *LmUAP2* dsRNA on *UAP* transcript levels in *L. migratoria* by RT-qPCR. The fifth-instar nymphs on day 3 were injected with ds*GFP*, ds*LmUAP1* or ds*LmUAP2*, respectively. The silencing efficiency of the two *LmUAP* genes was evaluated at 24 h and 48 h after the injection of dsRNA as shown in (A) and (B). The genes encoding β-actin and GAPDH were used as internal controls. Data are expressed as means ± SD of three biological replications. ** Shows significant differences between the nymphs injected with ds*LmUAP1* or ds*LmUAP2* and those injected with ds*GFP* (*P*<0.01, *t*- test); NS indicates no significant difference between the two groups.

To further analyze physiological significance of *LmUAP1* and *LmUAP2*, two specific dsRNAs targeting the most divergent regions of the two *LmUAP* genes were synthesized and injected into second- and fifth-instar nymphs. Total RNA was isolated at 24 and 48 h after dsRNA injection in fifth-instar nymphs (3- to 4-day-old). cDNA templates from above samples were used to examine the silencing efficiency by using RT-qPCR. The data were normalized with the genes encoding β-actin and GAPDH. As shown in Fig. 7, the expressions of both *LmUAP1* and *LmUAP2* were down-regulated at the time of detection, and the injection of *LmUAP1* or *LmUAP2* dsRNA only repressed the transcript level of the targeted *LmUAP* gene without reducing that of other *LmUAP* gene.

The effect of gene-specific silencing for each of the two *LmUAP* genes was examined by observing morphological changes or developmental arrest. When early second- and fifth-instar nymphs (both 1-day-old) were injected with *LmUAP1* dsRNA, these insects generally ceased feeding and were virtually unresponsive to physical touch. Nymphal mortalities reached at 100% about two days after the injection. In contrast, control nymphs fed actively and responded to the touch by vigorously wiggling or writhing.

**Figure 8:**
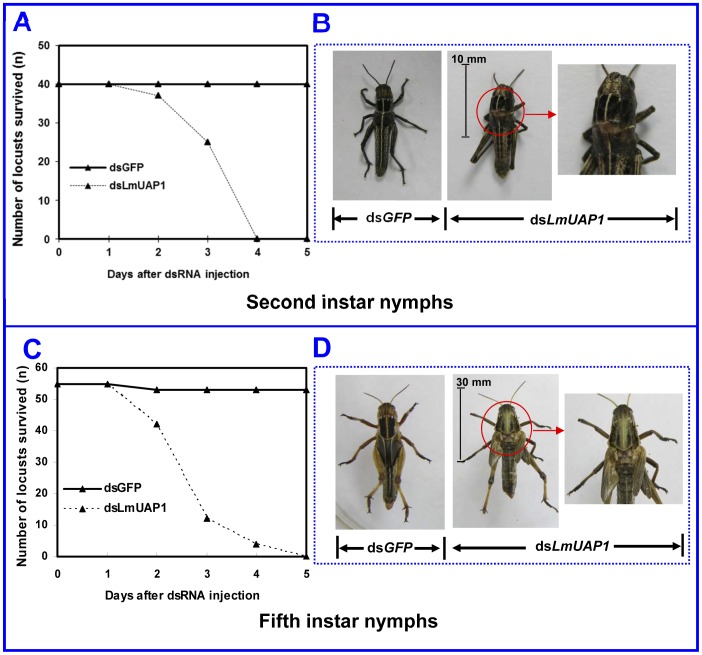
Effect of *LmUAP1* dsRNA on the development of *L. migratoria*. (A) and (B) show the survivorships and representative phenotypes of the nymphs after the ds*LmUAP1* and ds*GFP* injection into the second-instar nymphs on day 3–4. A total of 40 individuals were used in ds*GFP* or ds*LmUAP1* injection. The injection of *LmUAP1* dsRNA resulted in about 100% arrested individuals with only a slight split of the old cuticle (indicated by arrow). (C) and (D) show the survivorships and representative phenotypes of the nymphs after the ds*LmUAP1* and ds*GFP* injection into the fifth-instar nymphs on day 3–4. Total 55 individuals were used in ds*GFP* and ds*LmUAP1* injection, respectively. The similar phonotype was obtained as that in the second-instar nymphs.

When middle second- and fifth-instar nymphs (both 3- to 4-day-old) were injected with *LmUAP1* dsRNA, 100% of the insects died during the molting process (Fig. 8A, 8C). As shown in Fig. 8B and 8D, the dorsal split was typically initiated and the new cuticle was clearly visible, but the insects were unable to shed the old cuticle from their bodies. In contrast, when second- and fifth-instar nymphs on day 1 or day 3–4 were injected with *LmUAP2* dsRNA, they can undergo normal molting and development. Similarly, the mortality of the control group injected with ds*GFP* was only 5% and all the surviving insects can successfully molt to the next developmental stage.

## Discussion

UDP-GlcNAc, the active form of GlcNAc, is used not only during the biosynthesis of *O*- or *N*-linked oligosaccharides and the formation of GPI anchor but also as the substrate for the biosynthesis of chitin [6], [8], [9]. Commonly, UAP catalyzes the formation of UDP-GlcNAc from UTP and *N*-acetylglucosamin-1-phosphate. However, this enzyme has not been well studied, especially in eukaryotic systems. Here, we report for the first time the molecular characteristics and functions of UAPs from a typical hemimetabolous insect, *L. migratoria*.

Two full-length *UAP* cDNAs were discovered by searching the *L. migratoria* transcriptome database and confirmed by cDNA cloning and sequencing. The deduced enzymes of LmUAP1 and LmUAP2 show very similar theoretical molecular masses and pIs (slightly acidic). Both LmUAP1 and LmUAP2 contain the identical 17 amino acid residues, which appear to be important for substrate binding. Interestingly, all the arthropod UAPs have a cysteine at the position corresponding to 329 in LmUAP1, but in HsUAPB of *Homo sapiens* and CeUAP of *Caenorhabditis elegans*, this residue is replaced by an alanine.

LmUAP1 and LmUAP2 share 70% sequence identity at the amino acid level. This degree of amino acid sequence conservation is greater than those among insect UAP orthologs. LmUAP1 and LmUAP2 show the highest identities (63% and 58%, respectively) to the dipteran *Culex quinquefasciatus* UAP orthologs. Besides *L. migratoria*, *T. castaneum* is another insect species known to possess two different *UAP* genes which are located on different chromosomes and under the control of different promoters [15]. The nematode *C. elegans* also processes two *UAP* genes. CeUAP1 has additional 93 amino acid residues at the N-terminus compared with CeUAP2, but the remaining sequences share 94% identity at the amino acid level. In *D. melanogaster* and *H. sapiens*, two splice variants of *UAP* gene were found (named UAPA and UAPB in Fig. 3) [16], [24], [25]. Since most insects have a single *UAP* gene, the presence of two genes in *L. migratoria* and *T. castaneum* may indicate that the two *UAP* paralogs have derived from a relatively recent gene duplication event. Indeed, phylogenetic analysis of all known UAP proteins indicated that the two UAPs in *L. migratoria* or *T. castaneum* are first tightly clustered, and then clustered with the UAPs from other insect species. The UAPs of mammals appear close relationship with those of insects, whereas the UAPs from yeast and nematode are much less related to those of mammals and insects (Fig. 3).

In our previous work, we studied two chitin synthase genes (*LmCHS1* and *LmCHS2*) in *L. migratoria*. Our results indicate that the two genes have distinct tissue-specific expression patterns and functions in synthesizing chitin of the cuticle and PM [19], [20]. It would be also interesting to see whether there are any differences in expression patterns and biological functions between *LmUAP1* and *LmUAP2*.

For the tissue-dependent expression patterns, the highest expression of *LmUAP1* was found in the integument, whereas the highest expression of *LmUAP2* was found in the fatbodies of fifth-instar nymphs (Fig. 4). *LmUAP1* was also highly expressed in other chitin-containing tissues such as foregut and wings. In contrast, *LmUAP2* showed relatively low expressions in these tissues. Similarly, *TcUAP1* is also expressed in larval cuticle-forming tissues such as integument, hindwing and elytron as well as midgut in *T. castaneum*. The different expression patterns of *LmUAP1* and *LmUAP2* suggest that *LmUAP1* may be a major contributor to chitin biosynthesis in all the chitin-containing tissues but both *LmUAP1* and *LmUAP2* have additional functions due to their expressions in a wide range of tissues.

The expression of the two *LmUAP* genes was remarkably different during the insect development, particularly during the stages of embryogenesis and nymphal development. High expression of *LmUAP1* was detected during early embryogenesis but the expression decreased significantly in the remaining stages of embryogenesis. This expression pattern is very similar to those of other genes responsible for chitin biosynthesis in *L. migratoria*, such as glutamine: fructose-6-phosphate aminotransferase (*Gfat*) and chitin synthase (*CHS*) (data unpublished). *LmUAP1* expressed during early embryogenesis strongly suggests that it is of maternal origin and related to processes occurring early in embryogenesis. Indeed, it has been found that chitin is synthesized in the serosal cuticle (SC) 11–13 h after egg deposition, which may play an important role in the desiccation tolerance for *Aedes aegypti* eggs [26]. Thus, it is possible that the high expression of *LmUAP1* in early embryo may facilitate the biosynthesis of chitin with a role in desiccation tolerance as *L. migratoria* eggs are also tolerant to desiccation. In contrast, *LmUAP2* was expressed in similar amounts during the embryogenesis expect for its higher expression in 5-day-old eggs (Fig. 5), which implies that *LmUAP2* may have more broad functions, such as protein phosphorylation. Further work is needed to address biological function of *LmUAP2* in *L. migratoria*.

During the fourth- to fifth-instar nymphal development, the expression of *LmUAP1* in the integument was periodically repeated at each molting cycle. The transcript level of *LmUAP1* was high after molting, low during the inter-molting phases and then elevated again before the next molt. A similar phenomenon has been observed for *CHS1* in the larval or nymphal stage of *T. castaneum*, *Manduca sexta*, *Ostrinia furnacalis* and *Nilaparvata lugens* [27]–[30]. Considering the described ecdysone titer showing three peaks at 24, 64 and 104–120 h in fifth-instar nymphs [31], this distinct expression pattern may suggest that *LmUAP1* is involved in an ecdysone-dependent regulatory pathway. This phenomenon has been observed in *D. melanogaster,* in which *DmUAP* expression can be altered by the molting hormone 20-hydroxyecdysone [32]. Furthermore, the consistent expression of *LmUAP1* during the adult stage may be related to potential additional roles of this protein. However, the expression of *LmUAP2* was relatively unchanged during the nymphal and adult stages (Fig. 6). These results further support our notion that *LmUAP2* probably has different functions rather than chitin biosynthesis.

To explore the biological functions, RNAi was performed to silence each of the two genes in second- and fifth-instar nymphs. When second- and fifth-instar nymphs on day 1 were injected with *LmUAP1* dsRNA, all nymphs died about two days after the injection. As compared with the controls, insects injected with *LmUAP1* dsRNA hardly fed and were virtually unresponsive to touch. Such altered behaviors are similar to those of *L. migratoria* [20] and *T. castaneum* [33] whose *CHS2* genes were silenced by RNAi. In both species, the cessation of feeding associated with reduced chitin content in the midgut was observed. Thus, it is logically to expect that the injection of *LmUAP1* dsRNA may reduce chitin biosynthesis in the midgut because *UAP* is responsible for producing GlcNAc residues which are needed for chitin biosynthesis. This may explain why similar phenotypic responses are observed when *CHS2* and *UAP1* genes are silenced by RNAi in *L. migratoria* and other insect species.

When *LmUAP1* dsRNA was injected into second- and fifth-instar nymphs (on day 3–4), the insects can only partially split their old cuticle and died during the molting process (Fig. 8B, 8D). These phenotypes are almost identical to those of *T. castaneum* when *TcUAP1* dsRNA was injected in the last instar. Further examination has indicated that *TcUAP1*-specific RNAi lead to a significant reduction in chitin staining either in the elytra or the PM. It should be pointed out that RNAi of many chitin-remodeling related genes can lead to similar phenotypes as described above. These include *UAP1* as shown in this study and other studies in *T. castaneum* [33], trehalase 1 gene (*SeTre-1*) in *Spodoptera exigua* [34], chitin synthase 1 gene (*LmCHS1*) in *L. migratoria* [19], *N-*acetyl-hexosaminidase gene (*TcNAG1*) in *T. castaneum* [35], and chitin deacetylase gene (*TcCDA*) in *T. castaneum* [36]. All these suggest that these genes play a critical role in chitin biosynthesis and are essential for insect molting and development.

Nevertheless, when we injected *LmUAP2* dsRNA into second- and fifth-instar nymphs (either on day 1 or day 3–4), the insects were able to molt to next stage successfully and developed normally. Their adults showed normal fertility and longevity. This is distinctly different from those of insects subjected to RNAi for *LmUAP1*. RNAi of *TcUAP2* can affect larval growth or result in pupal paralysis, but there were no specific effects on the molting or PM structure in *T. castaneum*. Furthermore, after the injection of either of *TcUAP* dsRNAs in *T. castaneum* adult, mortality and depletion of fatbodies were clearly observed [15]. This is indirect evidence that *UAP* may play various biological functions besides chitin biosynthesis.

### Conclusion

We identified and characterized two different UDP*-N-*acetylglucosamine pyrophosphorylase genes (*LmUAP1* and *LmUAP2*) from *L. migratoria*. These genes showed distinct expression patterns during the insect development. *LmUAP1* plays a critical role in chitin biosynthesis and is essential for insect molting and development, whereas *LmUAP2* probably has different functions rather than chitin biosynthesis. Our results shed new light on different functions of *LmUAP1* and *LmUAP2* in the locust, and are expected to help researchers develop RNAi-based technologies targeting chitin biosynthesis for insect pest management.

## Supporting Information

Figure S1 **Ranking of the reference genes in different tissues of the fifth instar nymphs (2-day-old) of** ***L. migratoria*** **.** The expression stability of the potential reference genes was calculated by geNorm (A) and Normfinder (B). (A) geNorm gives an average expression stability measure (AESM) using stepwise exclusion of the least stable gene to organize candidate genes from the least (left) to the most stable (right). (B) Normfinder calculates a stability value which is proportional to the stability of the gene under different conditions.
(TIF)

Figure S2 **Ranking of the reference genes during the developmental stages of** ***L. migratoria*** **.** The expression stability of the potential reference genes were calculated by geNorm (A) and Normfinder (B).
(TIF)

Figure S3 **Ranking of the reference genes in the RNAi study in the fifth instar nymphs of** ***L. migratoria*** **.** The expression stability of the potential reference genes were calculated by geNorm (A) and Normfinder (B).
(TIF)

Table S1 (DOCX)
