# Supplementary figures and images for: Molecular and Functional Analysis of UDP-N-Acetylglucosamine Pyrophosphorylases from the Migratory Locust, Locusta migratoria
Source: PLoS One. 2013 Aug 19;8(8):e71970. doi: 10.1371/journal.pone.0071970 (PMC3747057; doi:10.1371/journal.pone.0071970)

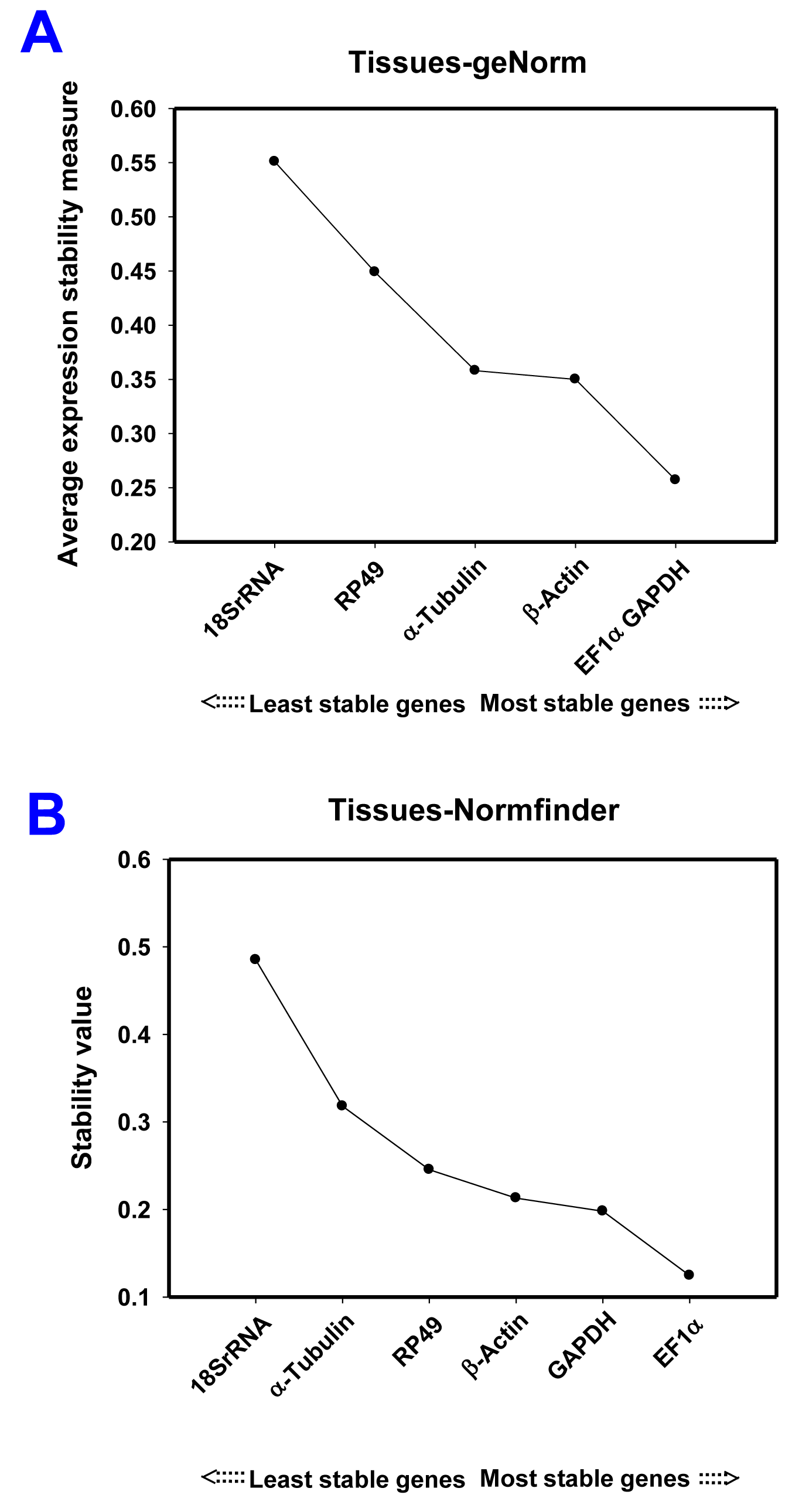

Supplement: Figure S1 — Ranking of the reference genes in different tissues of the fifth instar nymphs (2-day-old) of L. migratoria . The expression stability of the potential reference genes was calculated by geNorm (A) and Normfinder (B). (A) geNorm gives an average expression stability measure (AESM) using stepwise exclusion of the least stable gene to organize candidate genes from the least (left) to the most stable (right). (B) Normfinder calculates a stability value which is proportional to the stability of the gene under different conditions. (TIF) [file pone.0071970.s001.tif]

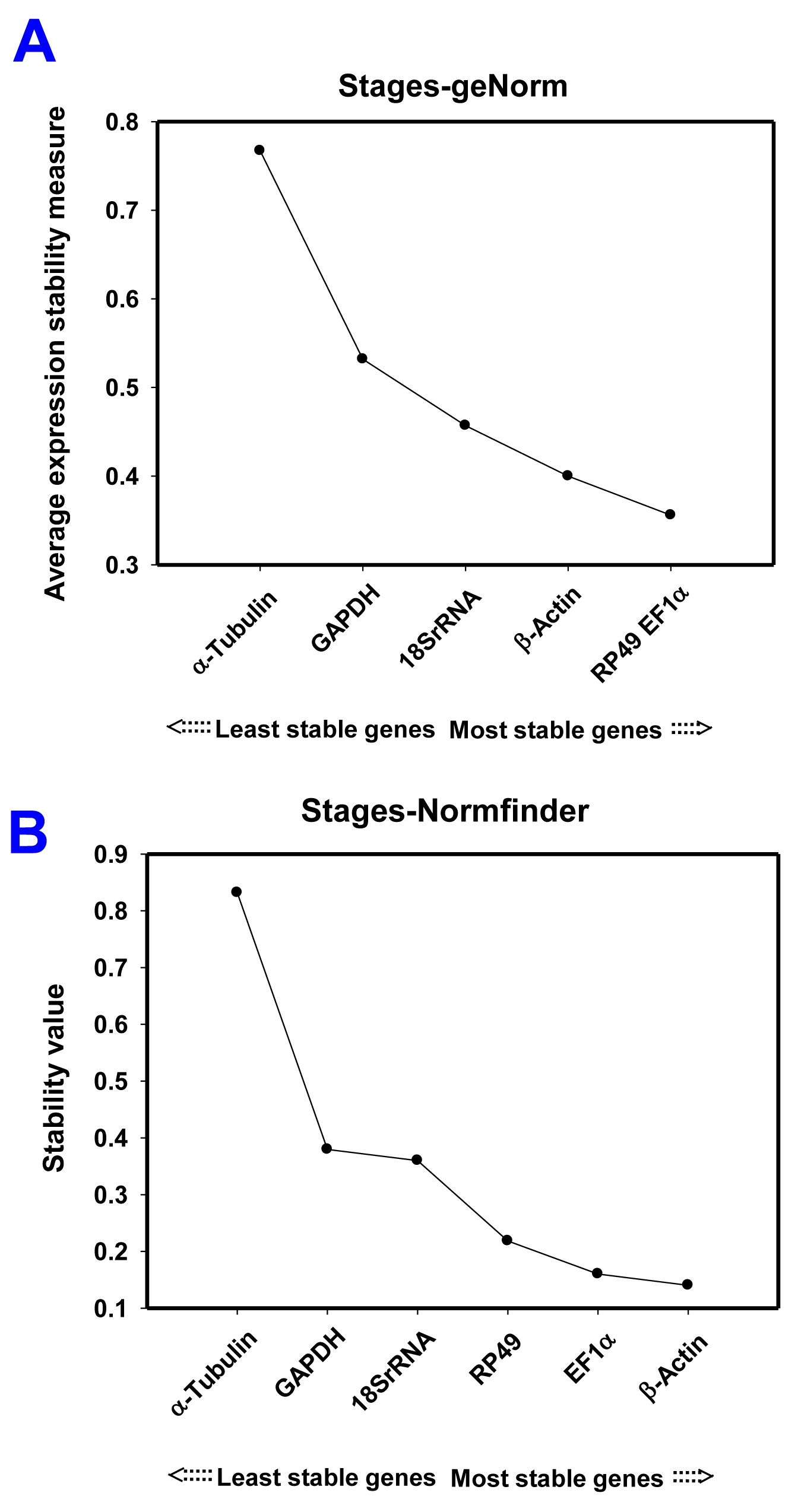

Supplement: Figure S2 — Ranking of the reference genes during the developmental stages of L. migratoria . The expression stability of the potential reference genes were calculated by geNorm (A) and Normfinder (B). (TIF) [file pone.0071970.s002.tif]

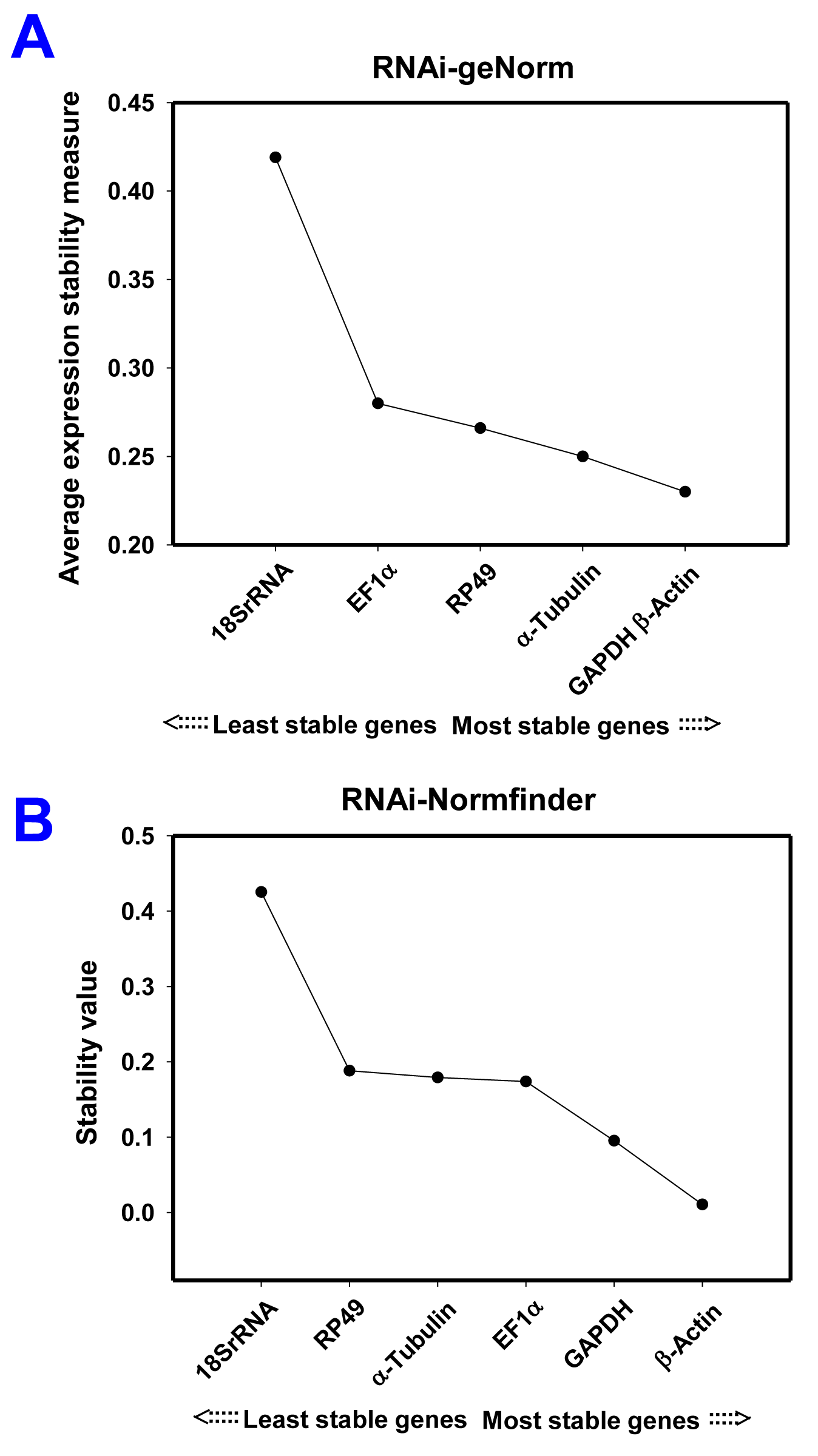

Supplement: Figure S3 — Ranking of the reference genes in the RNAi study in the fifth instar nymphs of L. migratoria . The expression stability of the potential reference genes were calculated by geNorm (A) and Normfinder (B). (TIF) [file pone.0071970.s003.tif]
