# Supplementary material for: Molecular and Functional Analysis of UDP-N-Acetylglucosamine Pyrophosphorylases from the Migratory Locust, Locusta migratoria
Source: PLoS One. 2013 Aug 19;8(8):e71970. doi: 10.1371/journal.pone.0071970 (PMC3747057; doi:10.1371/journal.pone.0071970)
Supplement: Table S1 — (DOCX) [file pone.0071970.s004.docx]

**Table S1.** Primers used to identify and validate the most stably expressed reference genes in this study.

| **Gene symbol** | **Gene name** | **Primer sequence (5’-3’)** | **Product size (bp)** | **Primer efficiency** |
| --- | --- | --- | --- | --- |
| *β-actin* | Beta actin | F: cgaagcacagtcaaagagaggta  R: gcttcagtcaagagaacaggatg | 156 | 98.2% |
| *EF1α* | Elongation factor 1α | F: AACATCGTCGTCATTGGTCA  R: GTTCAGCCTTCAGCTTGTCC | 175 | 99.4% |
| *GAPDH* | Glyceraldehyde-3-phosphate dehydrogenase | F: AGGCCAAGGTCAAGGAAGCT  R: GAATGGCAGTCACCAATGAAGTC | 120 | 92.2% |
| *RP49* | Ribosomal protein 49 | F: CGCTACAAGAAGCTTAAGAGGTCAT  R: CCTACGGCGCACTCTGTTG | 168 | 96.3% |
| *α-Tubulin* | Alpha tubulin | F: TGGTGTCCAACTGGTTTCAA  R: CGCTCTCTTGGCATACATCA | 174 | 96.5% |
| *18SrRNA* | 18S ribosome RNA | F: CTGAGAAACGGCTACCACATC  R: ACCAGACTTGCCCTCCAAT | 171 | 95.6% |
